# Secure Record Linkage of Large Health Data Sets: Evaluation of a Hybrid Cloud Model

**DOI:** 10.2196/18920

**Published:** 2020-09-23

**Authors:** Adrian Paul Brown, Sean M Randall

**Affiliations:** 1 Centre for Data Linkage Curtin University Bentley Australia

**Keywords:** cloud computing, medical record linkage, confidentiality, data science

## Abstract

**Background:**

The linking of administrative data across agencies provides the capability to investigate many health and social issues with the potential to deliver significant public benefit. Despite its advantages, the use of cloud computing resources for linkage purposes is scarce, with the storage of identifiable information on cloud infrastructure assessed as high risk by data custodians.

**Objective:**

This study aims to present a model for record linkage that utilizes cloud computing capabilities while assuring custodians that identifiable data sets remain secure and local.

**Methods:**

A new hybrid cloud model was developed, including privacy-preserving record linkage techniques and container-based batch processing. An evaluation of this model was conducted with a prototype implementation using large synthetic data sets representative of administrative health data.

**Results:**

The cloud model kept identifiers on premises and uses privacy-preserved identifiers to run all linkage computations on cloud infrastructure. Our prototype used a managed container cluster in Amazon Web Services to distribute the computation using existing linkage software. Although the cost of computation was relatively low, the use of existing software resulted in an overhead of processing of 35.7% (149/417 min execution time).

**Conclusions:**

The result of our experimental evaluation shows the operational feasibility of such a model and the exciting opportunities for advancing the analysis of linkage outputs.

## Introduction

### Background

In the last 10 years, innovative development of software apps, wearables, and the internet of things has changed the way we live. These technological advances have also changed the way we deliver health services and provide a rapidly expanding information resource with the potential for data-driven breakthroughs in the understanding, treatment, and prevention of disease. Additional information from patient devices, including mobile phone and Google search histories [1], wearable devices [2], and mobile phone apps [3], provides new opportunities for monitoring, managing, and improving health outcomes in new and innovative ways. The key to unlocking these data is in relating details at the individual patient level to provide an understanding of risk factors and appropriate interventions [4]. The linking, integration, and analysis of these data has recently been described as *population data science* [5].

Record linkage is a technique for finding records within and across one or more data sets thought to refer to the same person, family, place, or event [6]. Coined in 1946, the term describes the process of assembling the principal life events of an individual from birth to death [7]. In today’s digital age, the capacity of systems to match records has increased, yet the aim remains the same: linking records to construct individual chronological histories and undertake studies that deliver significant public benefit.

An important step in the evolution of data linkage is to develop infrastructure with the capacity to link data across agencies to create enduring integrated data sets. Such resources provide the capability to investigate many health and social issues. A number of collaborative groups have invested in a large-scale record linkage infrastructure to achieve national linkage objectives [8,9]. The establishment of research centers specializing in the analysis of *big data* also recognizes the issue of increasing data size and complexity [10].

As the demand for data linkage increases, a core challenge will be to ensure that the systems are scalable. Record linkage is computationally expensive, with a potential comparison space equivalent to the Cartesian product of the record sets being linked, making linkage of large data sets (in the tens of millions or greater) a considerable challenge. Optimizing systems, removing manual operations, and increasing the level of autonomy for such processes is essential.

A wide range of software is currently used for record linkage. System deployments range from single desktop machines to multiple servers, with most being hosted internally to organizations. The functional scope of packages varies greatly, with manual processes and scripts required to help manage, clean, link, and extract data. Many packages struggle with large data set sizes.

Many industries have moved toward cloud computing as a solution for high computational workloads, data storage, and analytics [11]. An overview of cloud computing types and service models is shown in Table 1. The business benefits of cloud computing include usage-based costing, minimal upfront infrastructure investment, superior collaboration (both internally and externally), better management of data, and increased business agility [12]. Despite these advantages, uptake by the record linkage industry has been slow. One reason for this is that the storage of identifiable information on cloud infrastructure has been assessed as high risk by data custodians. Although security in cloud computing systems has been shown to be more robust than some in-house systems [13], the media reporting of data breaches has created an impression of insecurity and vulnerability [14]. A culture of risk aversion leaves the record linkage units with expensive, dedicated equipment and computing resources that require managing, maintaining, and upgrading or replacing regularly.

**Table 1:** Overview of cloud computing types and service models.

Name		Description	
**Types of cloud computing**			
	Public		All computing resources are located within a cloud service provider that is generally accessible via the internet.
	Private		Computing resources for an organization that are located within the premises of the organization. Access is typically through local network connections.
	Hybrid		Cloud services are composed of some combination of public and private cloud services. Public cloud services are typically leveraged in this situation for increasing capacity or capability.
**Service models**			
	IaaS^a^		The provider manages physical hardware, storage, servers, and virtualization, providing virtual machines to the consumer.
	PaaS^b^		In addition to the items managed for IaaS, the provider also manages operating systems, middleware, and platform runtimes. The consumer leverages these platform runtimes in their own apps.
	SaaS^c^		The provider manages everything, including apps and data, exposing software endpoints (typically as a website) for the consumer.

^a^IaaS: Infrastructure as a Service.

^b^PaaS: Platform as a Service.

^c^SaaS: Software as a Service.

To leverage the advantages of cloud computing, we need to explore operational cloud computing models for record linkage that consider the specific requirements of all stakeholders. In addition, linkage infrastructure requires the development and implementation of robust security and information governance frameworks as part of adopting a cloud *solution*.

### Related Work

Some research on algorithms that address the computational burden of the comparison and classification tasks in record linkage has been undertaken. Most work on distributed and parallel algorithms for record linkage is specific to the MapReduce paradigm [15], a programming model for processing large data sets in parallel on a cluster. Few sources detail the comparison and classification tasks themselves, with the focus on load balancing algorithms to address issues associated with data skew. These works attempt to optimize the workload distribution across nodes while removing as many true negatives from the comparison space as possible [16-19]. Load balancing algorithms typically use multiple MapReduce jobs and different indexing methods to tackle the data skew problem. Indexing methods include standard blocking [17,18], density-based blocking [16], and locality sensitive hashing (LSH) [20], with varying success in optimizing the workload distribution.

Pita et al [21] have built on the MapReduce-based work and demonstrated good performance and quality using a Spark-based workflow for probabilistic linkage. Spark was chosen for in-memory processing, ease of programming, scalability, and the new resilient distributed data set model. Like MapReduce, Spark continues to be used to address the issues with linkage and data skew on larger data sets. Spark solutions for full entity resolution are being developed, with different indexing techniques used to address workload distribution. The SparkER tool by Gagliardelli et al [22] uses LSH, meta-blocking, and a block purging process to remove high-frequency blocking keys. Mestre et al [23] presented a sorted neighborhood implementation with an adaptive window size, which uses three Spark transformation steps to distribute the data and minimize data skew.

Outside of the Hadoop ecosystem, which MapReduce and Spark are a part of, there have been some efforts to address the linkage of larger data sets through other parallel processing techniques. Sehili et al [24] presented a modified version of PPJoin, called P4Join, that can parallelize record matching on graphics processing units (GPUs), claiming an execution time improvement of up to 20 times. Despite its potential for significant improvements in runtime performance, there has not been any further work published on P4Join using larger data sets or on clusters of GPU nodes. More recently, Boratto et al [25] evaluated a hybrid algorithm using both GPUs and central processing units (CPUs) with much larger data sets. Although restricted to single (highly specified) machines, these evaluations show promise provided that the approach can be applied within a compute cluster. Again, there is not yet any further work available.

The blocking techniques used in these studies are based on the same techniques used for traditional probabilistic and deterministic linkages [15]. There are many blocking techniques used in these conventional approaches to record linkages that reduce the comparison space significantly, even when running a linkage on a single machine [26]. However, these approaches become inefficient as data set sizes become larger. They also come with a trade-off; the creation of blocks that reduce the comparisons required for linkage will inevitably reduce the coverage of true matches, resulting in more missed matches.

Much of the work in distributed linkage algorithms is focused on performance, often at the expense of linkage accuracy. Adapting these blocking techniques to distribute workload across many compute nodes has reduced the comparisons efficiently. Unfortunately, this increased efficiency has impacted the accuracy further, reducing comparisons at the expense of missing more true matches. There is still a trade-off between performance and accuracy, and further work is required to address it.

### Data Flow and Release for Record Linkage

As data custodians are responsible for the use of their data, researchers must demonstrate to custodians that all aspects of privacy, confidentiality, and security have been addressed. The release of personal identifiers for linkage can be restricted, with privacy regulations such as the Health Insurance Portability and Accountability Act Privacy Rules [27] or EU regulations [28] mandating the use of encrypted identifiers. Standard record linkage methods and software are often unsuitable for linkage based on encrypted identifiers. Privacy-preserving record linkage (PPRL) techniques have been developed to enable linkage on encrypted identifiers [29]. These techniques typically use Bloom filters to store encrypted identifiers, a probabilistic data structure that can be used to approximate the equality of two sets. The emergence of these PPRL methods means that data custodians are not required to release personal identifiers. The use of PPRL methods in operational environments is still in its infancy, with limited tooling available. Available software includes the proprietary LinXmart [30], an R package called PPRL developed by the German Record Linkage Center [31], LSHDB [32], LinkIT [33], and Secure Open Enterprise Master Patient Index [34]. There is little published data on how much these systems are used outside of the organizations that created them. PPRL is a key technology that greatly opens the acceptability of cloud solutions for record linkage.

### Record Linkage Process

**Figure 1:**
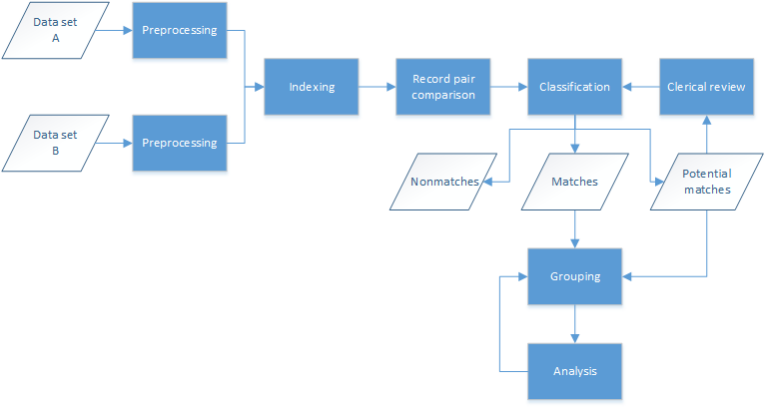
Typical data matching process.

Record linkage typically follows a standard process for the matching of two or more data sets, as shown in Figure 1. The data sets first undergo some preprocessing, a cleaning and standardization step to ensure consistency with the formatting of fields across data sets. The next step (indexing) attempts to reduce the number of record-level comparisons required (the latter often referred to as the comparison space), removing comparisons that are most likely to be false matches. The indexing step typically groups data sets into overlapping blocks or clusters based on sets of field values and can provide up to 99% reduction in the comparison space. Record pair comparisons occur next, within the blocks or clusters determined during the indexing step; this comparison step is the most computationally expensive and often requires large data sets to be broken down into smaller subsets. Classification of the record pairs into matches, nonmatches, and potential matches results in groups of entities (or individuals) based on the match results. Potential matches can be assessed manually or through special tooling to determine whether they should be classified as matches or nonmatches. A common approach to grouping matches is to merge all records that link together into a single group; however, different approaches can be used to reduce linkage error [35]. Analysis of the entity groups is the last step, where candidate groups are clerically reviewed to determine if and how the records in these groups should be regrouped.

This paper presents 2 contributions to record linkage. First, it offers a model for record linkage that utilizes cloud computing capabilities while providing assurance that data sets remain secure and local. Lessons learned from many real-world record linkage projects, including several PPRL projects, have been instrumental in the design of this cloud model [30,36,37]. Second, the use of containers to distribute linkage workloads across multiple nodes is presented and evaluated within the cloud model.

## Methods

### Design of a Cloud Model for Record Linkage

The standard record linkage process relies on one party (known as the trusted third party [TTP]) having access to all data sets. Handling records containing identifiable data requires a sound information governance framework with controls in place that manage potential risks. Even with a well-managed information security system in place, access to some data sets may still be restricted. The TTP also requires infrastructure that can help manage data sets, matching processes and linkage key extractions over time. As the number and size of data sets grow, the computational needs and storage capacity must grow with it. However, the computation requirements for data linkage are often sporadic bursts of intense workloads, leaving expensive hardware sitting idle for extended periods.

Dedicated data linkage units in government and academic institutions exist across Australia, Canada, and the United Kingdom, acting as trusted third parties for data custodians. These data linkage units were established from the need to link data for health research at the population level. Some data linkage units are involved in the linkage of other sectors such as justice; however, the primary output of these organizations is linked data for health research. It is essential that a cloud model for record linkage takes into account the linkage practices and processes that have been developed by these organizations.

Our cloud model for record linkage addresses the limitations of data release and the computational needs of the linkage process. Data custodians and linkage units retain control of their identifiable information, while the matching of data sets between custodians occurs within a secure cloud environment.

### Tenets of the Record Linkage Cloud Model

The adopted model was founded on 3 overarching design principles:

1. *The privacy of individuals in the data is protected.* One of the most important responsibilities for data custodians and linkage units is information security. Data sets contain private, and often sensitive, information on people, and it is vital that appropriate controls are in place to mitigate any potential risks. Some data sets have restrictions on where they can be held, requiring them to be kept local and protected. All computation and storage within the cloud infrastructure must be done on privacy-preserved versions of these data sets.
2. *Computation and storage are outsourced to* the cloud infrastructure. Computation requirements for data linkage are often sporadic bursts of intense workloads, followed by periods of low use or even inactivity. The ability to provision resources for computation as and when required means you only pay for what you use. This computation is generally associated with large sets of input and output data, so it makes sense to keep these data as close to the computation as possible. Storage may not necessarily be cheap, but many cloud computing providers guarantee high levels of durability and availability, with encryption and redundancy capabilities.
3. *Cloud platform services are used over infrastructure services.* Once data are stored within a cloud environment, additional Platform as a Service offerings for analysis of the data should be leveraged. These are managed services over the top of infrastructure services (such as virtual machines) and can be started and stopped as needed.

### High-Level Architectural Model

**Figure 2:**
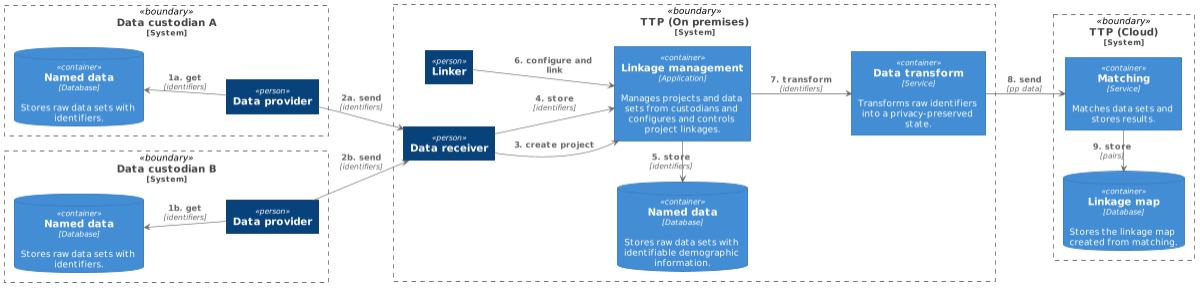
Hybrid cloud trusted third party model. PP: privacy-preserved; TTP: trusted third party.

Not all storage and computation can be performed within a cloud environment without impacting privacy; the storage of raw identifiers (such as name, date of birth, and address) must often remain on-premises. The heavy-computational workloads for record linkage, the record pair comparisons and classification, are therefore undertaken on privacy-preserved versions of these data sets. These privacy-preserved data sets must be created on premises and uploaded to cloud storage. The remainder of the linkage process continues within the cloud environment. However, some parts of the classification and analysis steps may be done interactively by the user from an on-premises client app, annotating results from cloud-based analytics with locally stored details (ie, identifiers). An overview of the components and data flows involved in the hybrid TTP model is shown in Figure 2. This model satisfies our cloud model tenets and provides the linkage unit with the ability to scale their infrastructure on-demand. The matching (classification) component can utilize scalable platform services available by the cloud provider to match large privacy-preserved data sets as required. All major cloud providers have platform services that can provide computation on-demand for the processing of big data. The linkage map persists as it contains no identifiable information and can also be analyzed using available cloud platform services.

**Figure 3:**
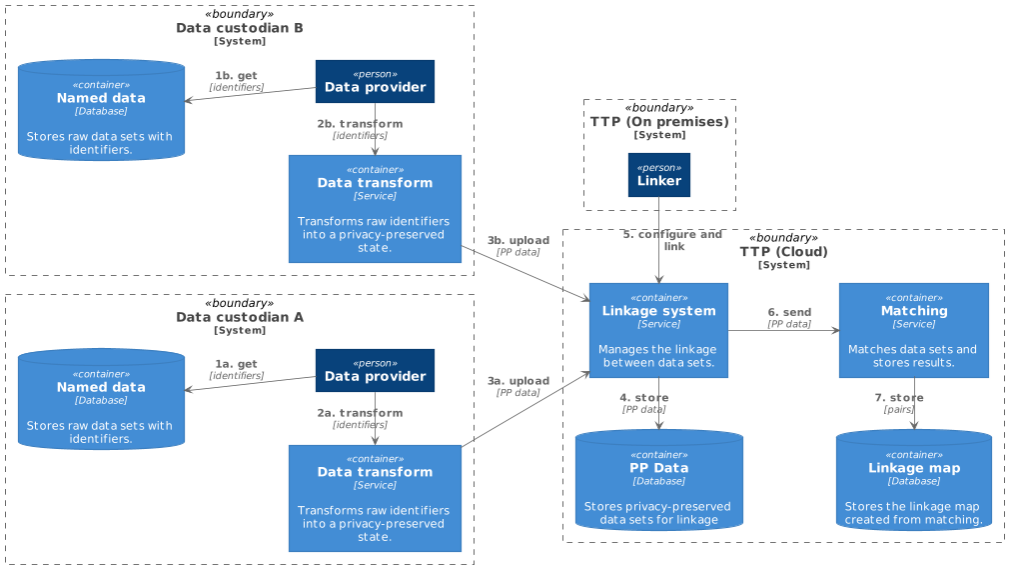
Full cloud trusted third party model. PP: privacy-preserved; TTP: trusted third party.

Keeping identifiers at the data custodian level (on-premises) while matching on privacy-preserved data within cloud infrastructure enables linkages of data sets *between* data custodians. This model does not require any raw identifiers to be released, and thus, a hybrid model is no longer necessary. The TTP can then be hosted fully in the cloud, as shown in Figure 3. There are 2 immediate ways to achieve this: either one of the custodians manages the cloud infrastructure themselves or an independent third party controls it and provides it as a service to all custodians. A custodian could act as a TTP for all custodians involved in the linkage if this is acceptable to the parties involved. Otherwise, it may be more amenable to go with an independent TTP.

Although the full cloud TTP model may be useful in some situations, it is unlikely that this would be a desirable model with the dedicated data linkage units. Processes in cleaning, standardization, and quality analysis with personal identifiers have developed and matured over many years. Switching to a model where they no longer have access to personal identifiers would affect the accuracy of the linkage and ultimately the quality of the health research that used the linked data. The hybrid model replaces only the matching component, allowing many existing linkage processes to remain.

### Scaling Computation-Heavy Workloads

Record pair comparison and classification tasks are the most computationally intensive tasks in the linkage process, although they are heavily affected by the indexing method used. The single process limitation of most linkage apps makes it difficult to cater to increasingly large data sets, regardless of indexing. Increasing memory and CPU resources for these single-process apps provides some ability to increase capacity, but this may not be sustainable in the longer term.

Although MapReduce appears to be a promising paradigm for addressing large-scale record linkage, 2 issues emerge. First, they consider only the creation of record pairs, whether matches or potential matches, without any thought as to how these record pairs are to come together to form entity groups. The grouping task is also an important part of the data matching process, and the grouping method used can significantly reduce matching errors [35]. Second, MapReduce algorithms do not appear to be readily used, if at all, within an operational linkage environment. Organizational change can be slow, and there is much investment in the existing matching algorithms and apps currently used. It may be operationally more acceptable to continue using these apps where possible.

The comparison and classification tasks of the record linkage process are an embarrassingly parallel problem if the indexing task can produce disjoint sets of record pairs (blocks) for comparison. With the rapid uptake of containerization and the availability of container management and orchestration capability, a viable option for many organizations is to reuse existing apps deployed in containers and run in parallel. Matching tasks on disjoint sets can be run independently and in parallel. The matches and potential matches produced by each matching task can, in turn, be processed independently by grouping tasks. The number of sets that are run in parallel would then only be limited by the number of container instances available.

Indexing solutions are imperfect on real-world data; however, producing disjoint sets for matching is difficult without an unacceptable drop in *pairs completeness* (a measure of the coverage of true positives). There is inevitably some overlap between blocks, as multiple passes with different blocking keys are typically used to ensure accurate results. This overlap prevents independent processing and can be handled in 1 of the 2 ways: (1) the blocks of pairs for classification can be calculated in full before duplicates are removed and the classification task can be run or (2) duplicate matches and potential matches are removed following the classification task. The main disadvantage of option 1 is that this requires a potentially massive set of pairs to be created upfront, as the comparison space is typically orders of magnitude larger than the set of matches and potential matches. Many linkage systems combine their indexing and classification tasks for efficiency, and it is often easier to ignore duplicate matches until completion. The disadvantage of option 2 is that overlapping block sets result in overlapping match sets, preventing the independent grouping of matches from each classification task.

Regardless of the indexing method used to reduce the comparison space for matching, the resulting blocks require grouping into manageable size bins that can be distributed to parallel tasks. A *bin*, therefore, refers to a subset of record pairs grouped together for efficient matching. Block value frequencies are calculated across data sets and used to calculate the size of the total comparison space. Records from these data sets are then copied into separate bins such that each bin has a comparison space of approximately equal size to every other bin.

Using this method, the comparison and classification of each bin are free to be executed on whatever compute capability is available. A managed container cluster is an ideal candidate; however, the container’s resources (CPU, memory, and disk) and the bin characteristics (eg, maximum comparison space) need to be carefully chosen to ensure efficient resource use.

### Development and Experimental Evaluation of the Prototype

An evaluation of the hybrid cloud linkage model was conducted through the deduplication of different sized data sets on a prototype system. The experiments were designed to evaluate parallel matching using an existing matching app on a cluster of containers; to measure encryption, transfer, and execution times; and to assess the remote analysis of the matching pairs created.

A prototype system was developed with the on-premises component running on Microsoft Windows 10 and the cloud components running on Amazon Web Services (AWS). The prototype focused on the matching part of the linkage model and utilized platform services where available. These services are described in Table 2.

**Table 2:** Amazon Web Services used.

AWS^a^	Description
S3	Provides an object (file) storage service with security, scalability, and durability.
Glue	A fully managed extract, transform, and load service, providing table definition, schema discovery, and cataloging. Used in conjunction with S3 to expose cataloged files to other AWS services.
Step function	A managed state machine with workflows involving other AWS. The output of a step that uses a particular service can then be used as the input for the next step.
Batch	A fully managed service for running batches of compute jobs. Compute resources are provisioned on-demand.
Athena	An interactive query service for analyzing data in S3 using standard Structured Query Language.

^a^AWS: Amazon Web Services.

### Test Data

Three synthetic data sets were generated to simulate population-level data sets: 7 million records, 25 million records, and 50 million records. Although 7 million records may not necessarily represent a large data set, a 50 million record data set is challenging for most linkage units. The data sets were created with a deliberately large number of matches per entity to increase the comparison space and to challenge the matching algorithm.

Data generation was conducted using a modified version of the Febrl data generator [38], an open-source data linkage system written in Python. Frequency distributions of the names and dates of birth of the population of Western Australia were used to generate the synthetic data sets. Randomly selected addresses were sourced from Australia’s National Address File, a publicly available data set [39]. Each data set contained first name, middle name, last name, date of birth, sex, address, and postcode fields. Each field had its own rate of errors and distribution of types of errors. These were based on previously published synthetic data error rates, deliberately set high to challenge matching accuracy [40]. Type of errors included replacement of values, field truncation, misspellings, deletions, insertions, use of alternate names, and values set to missing. Records had anywhere between zero to many thousands of duplicates within the data sets.

All available fields were used for matching in a probabilistic linkage. Two separate blocks were used: first name initial and last name Soundex, and date of birth and sex. Each pair output from the matching process included two record IDs, a score, a block (strategy) name, and the individual field-level comparison weights used to calculate the score.

### Experiments

The on-premises component first transformed data sets containing named identifiers into a privacy-preserved state using Bloom filters. String fields were split into bigrams that were hashed 30 times into Bloom filters 512 bits in length. Numeric fields (including the specific date of birth elements) were cryptographically hashed using hash-based message authentication code Secure Hash Algorithm 2 (SHA2). These privacy-preserved data sets were compressed (using gzip) before being uploaded to Amazon’s object storage, S3. A configuration file was also uploaded, containing the necessary linkage parameters required for the probabilistic linkage. An AWS step function (a managed state machine) was then triggered to run through a set of tasks to complete the deduplication of the file as defined in the parameter file.

All step function tasks used on-demand resource provisioning for computation. A compute cluster managed by AWS Batch was configured with a maximum CPU count of 40 (10×c4.xlarge instance type). Each container was configured with 3.5 GB RAM and 2 CPUs, allowing up to 20 container instances to run at any one time.

**Figure 4:**
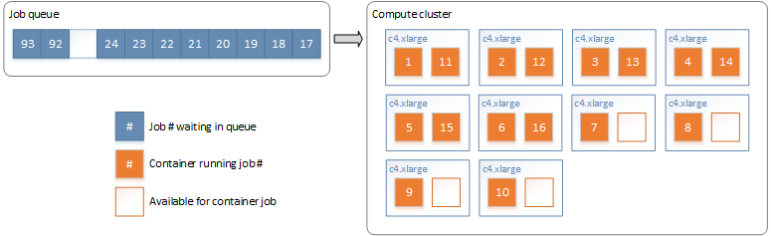
Matching jobs running on compute cluster (one job per bin).

The first task ran as a single job, splitting the file into many bins of approximately equal comparison space, using blocking variables specified in the configuration file. By splitting on the blocking variables, the comparison space for the entire linkage remains unchanged. Each bin was stored in an S3 location with a consistent name suffixed with a sequential identifier. The second task ran a node array batch job, with a job queued for each bin to run on the compute cluster. Docker containers running a command-line version of the LinXmart linkage engine were executed on the compute nodes to deduplicate each bin independently. AWS Batch managed the job queue, assigning jobs to available nodes in the cluster, as shown in Figure 4.

LinXmart is a proprietary data linkage management system, and the LinXmart linkage engine was used because of our familiarity with the program and its ability to run as a Linux command-line tool. It accepts a local source data set and parameter file as inputs and produces a single pairs file as output. There were no licensing issues running LinXmart on AWS in this instance, as our institution has a license allowing unrestricted use. This linkage engine could be substituted, if desired, for others that similarly produce record pair files. The container was bootstrapped with a shell script that downloaded and decompressed the source files from S3 storage, ran the linkage engine program, and then compressed and uploaded the resulting pairs file to S3 storage. Each job execution was passed a sequential identifier by AWS Batch, which was used to identify a source bin datafile to download from S3 and mark the resulting pair file to upload to S3.

**Figure 5:**
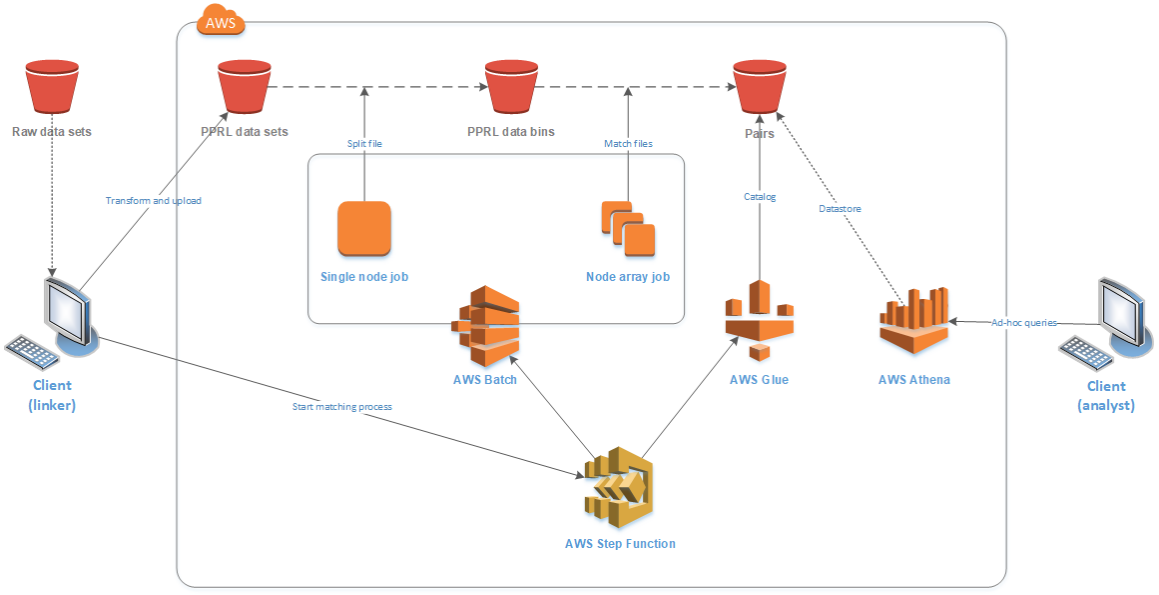
Prototype on Amazon Web Services. AWS: Amazon Web Services; PPRL: privacy-preserved record linkage.

The third step function task classified and cataloged all new pairs files, using AWS Glue, making them available for use by other AWS analytical services. The results for each original data set were then able to be presented as a single table, although the data itself were stored as a series of individual text files. The prototype’s infrastructure and data flow are shown in Figure 5.

Once the deduplication linkages were complete, the on-premises component of the prototype was employed to query each data set’s pair table. The queries were typical of those used following a linkage run: pair count, pair score histogram, and pairs within a pair score range. This query component used the AWS Athena application programming interface (API) to execute the queries, which used Presto (an open-source distributed query engine) to apply the ad hoc structured query language queries to the cataloged pairs tables.

## Results

### Design of a Cloud Model for Record Linkage

**Figure 6:**
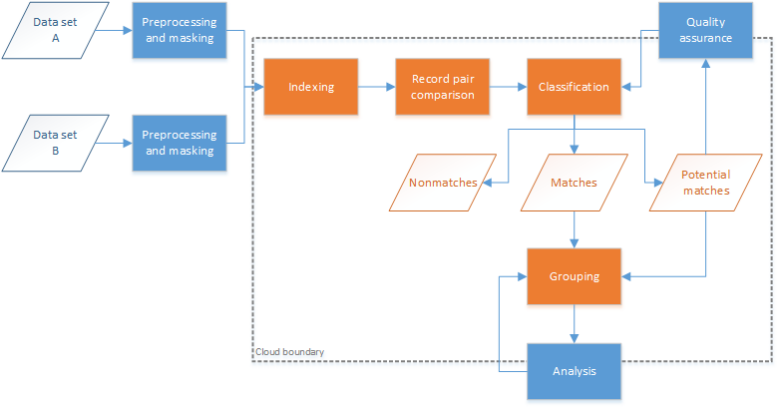
New cloud model data matching process.

The cloud model data matching process is shown in Figure 6. Essentially, every step in the record linkage process from indexing to group analysis is pushed to cloud infrastructure. Preprocessed data sets are transformed into a privacy-preserved state (masking) and uploaded to the cloud service for linking. The services within the cloud boundary now act as a TTP. The quality assurance and analysis steps sit on the boundary of the cloud as computation and query occur on the hosted cloud infrastructure, but the interactive analysis is performed by the analysis client on premises. If the analysis client has access to one or more of the raw data sets used in the linkage, these data can be annotated onto query results, giving the clerk more informed decisions and an experience to which they are accustomed.

**Figure 7:**
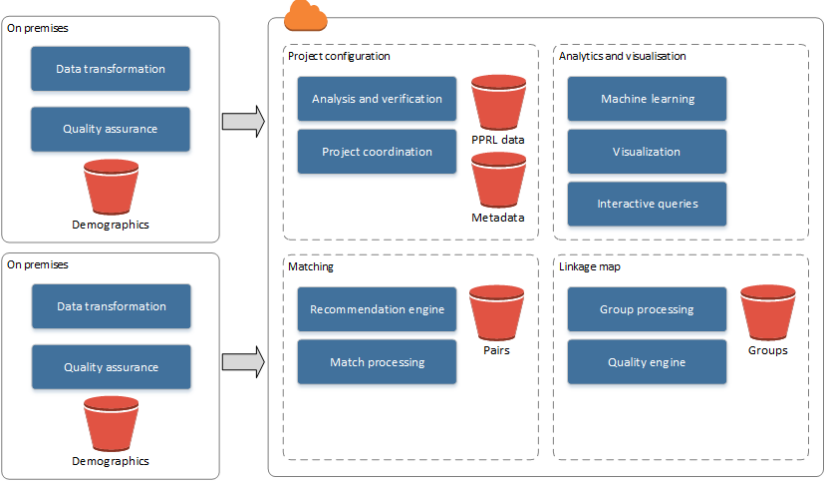
High-level architecture of record linkage cloud model. PPRL: privacy-preserved record linkage.

As shown in the high-level architectural model in Figure 7, the demographic data (containing personal identifiers) continue to remain on premises with the data custodian. Responsibilities of the data custodians are limited to data transformation and quality assurance management. The responsibilities of the cloud services are covered under 4 main categories: project configuration, matching, linkage map, and analytics and visualization. The project configuration includes the services required for coordinating projects within and across separate data custodians. Privacy-preserved data sets are stored here as well as metadata on the data sets as a result of analysis and verification performed on the uploaded data sets. The matching category includes all match processing (classification) and pairs output as well as services for providing recommendations on linkage parameters (such as *m* and *u* likelihood estimates for probabilistic linkages) for linkages between privacy-preserved data sets [41]. The linkage map category holds the entity group information, the map between individual records, and the group in which they belong. This category also contains services for processing and creating groups from pairs as well as quality estimation and analysis. Analytics and visualization contain all analytical services provided to the on-premises clients.

This model also allows computation to be pushed onto inexpensive, on-demand hardware in a privacy-preserving state while retaining the advantage of seeing raw identifiers during other phases of the linkage process (eg, quality assurance and analysis).

### Experimental Evaluation of the Prototype

**Figure 8:**
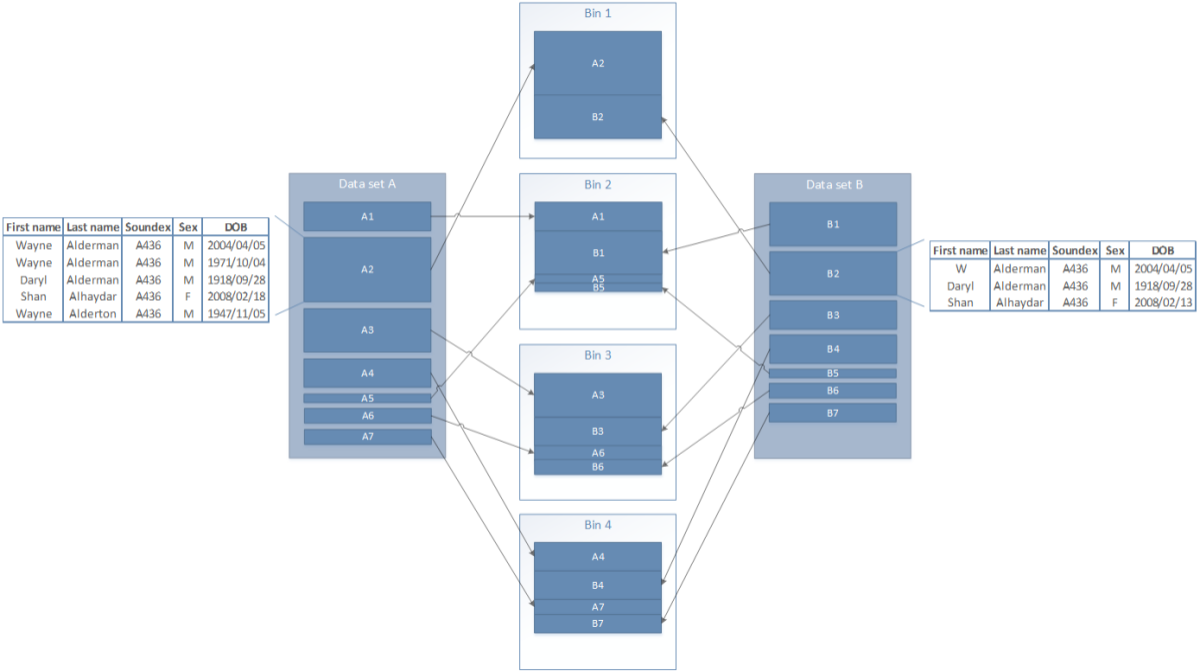
Datafiles split into independent bins (by Soundex block values) for matching. DOB: date of birth.

Each deduplication consisted of a single node job to split the data set into multiple bins, followed by a node array job for the matching of records within each bin. The split of data into bins is shown in Figure 8. In this example, all records with the same Soundex value will end up in the same bin.

The total comparison space was calculated using the blocking field frequencies in the data set. These frequencies represent the number of times each blocking field value occurs in the data, providing the ability to calculate the number of comparisons that will be performed for each blocking field value. First, the comparison space for each blocking field was calculated using the frequency of the value within the file. The total comparison space was the sum of each, and the bin count was determined by dividing this by the maximum desired comparison space for a single bin. The blocking field value with the largest comparison space was assigned to the first bin. The blocking field value with the next largest comparison space was assigned to the second bin. This process continued for each blocking field value, returning to the first bin when the end was reached. A file was created for each bin, which was then independently deduplicated. Blocking field values with a very high frequency are undesirable as they are usually less useful for linkage and are costly in terms of computation. Any blocking field value with a frequency higher than the maximum desired comparison space was discarded.

The total comparison space used for each data set, along with the bin count and pair count, is presented in Table 3. The two blocks used for the creation of separate bins for distribution across the processing cluster resulted in some duplication of comparisons and, thus, duplication of pairs.

**Table 3:** Comparison space and pairs created during classification.

Data set size (millions)	Comparison space, n	Bins, n	Total pairs, n	Unique pairs, n	Pairs files size (GB)
7	2,745,977,009	28	634,544,432	415,444,583	9
25	18,458,616,866	93	2,169,337,646	1,594,343,961	22
50	53,848,633,907	270	4,424,983,776	3,260,509,561	44

**Figure 9:**
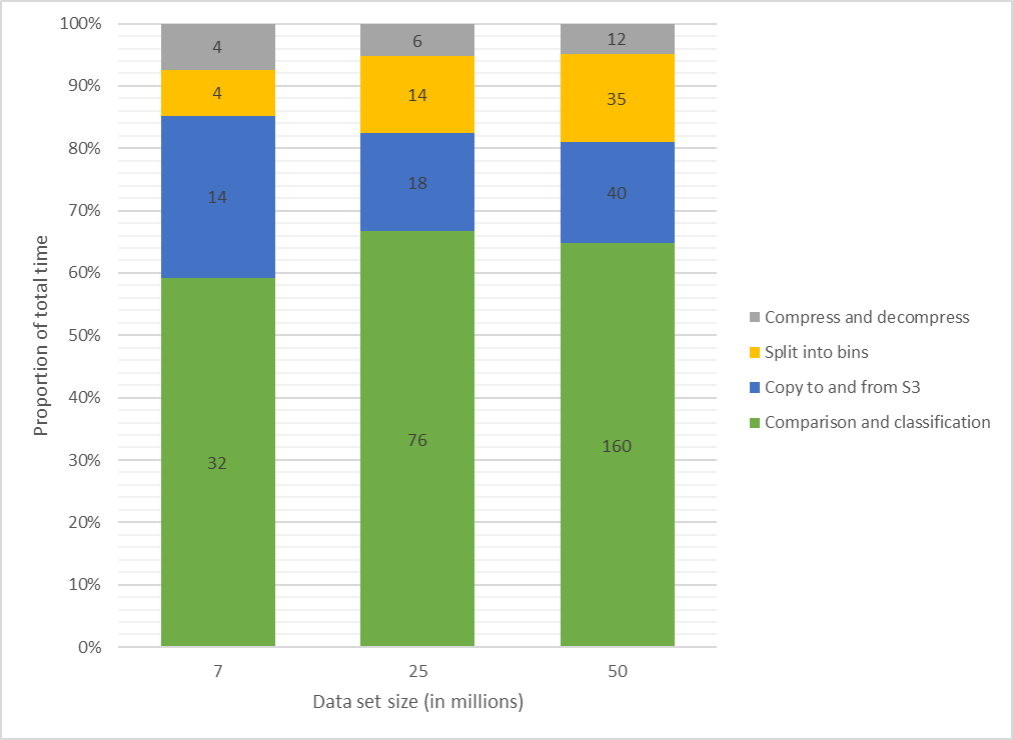
Task execution time (in minutes) and the proportion of total time.

Approximately 60% of the time was spent on comparison and classification by each container (Figure 9). Much of the time was spent managing data in and out of the container itself. Splitting a data set into bins for parallel computation took between 7% (4/54 minutes) and 14% (35/247 minutes) of the total task time, a reasonable sacrifice considering the scalability factor this gives for the classification jobs. Provisioning of the compute resources took between 2 and 4 min for each data set.

Running times of ad hoc queries on data sets are shown in Table 4; these were each executed 5 times on the client and run through the AWS Athena API. The mean execution time did not vary greatly across the differently sized data sets. With a simple count query taking around 25 seconds, there appears to be some initial setup time for provisioning the backend Presto cluster. This is expected and should not be considered an issue, particularly with all queries of the largest data set of 4.4 billion pairs taking less than 1 min to execute.

**Table 4:** Mean execution times for sample queries on full pairs set.

Data set size (millions)	Pairs count (millions)	Sample queries		
		Count (seconds)	Pair score histogram (seconds)	Fetch pairs in score range 15-16 (seconds)
7	635	26	52	51
25	2169	27	56	53
50	4424	24	52	54

In terms of costs associated with the use of AWS cloud services for our evaluation, there were 2 main types. First, the cost of on-demand processing, which is typically charged by the second. This totaled just over US $20 for the linkage processing used for all 3 data sets. The second is the cost of storage, which is charged per month. To retain the pairs files generated for all 3 data sets, it cost only US $2 per month. Querying data via the Athena service is currently charged at US $5 per terabyte scanned.

## Discussion

### Principal Findings

Our results show that an effective cloud model can be successfully developed, which extends linkage capacity into cloud infrastructure. A prototype was built based on this model. The execution times of the prototype were reasonable and far shorter than one would expect when running the same software on a single hosted machine. Indeed, it is likely that on a single hosted machine, the large data set (50 million) would need to be broken up into smaller chunks and linkages on these chunks run sequentially.

The splitting of data for comparison into separate bins worked well for distributing the work and mapped easily to the AWS Batch mechanism for execution of a cluster of containers. The creation of an AWS step function to manage the process from start to end was relatively straightforward. Step functions provide out-of-the-box support for AWS Batch. However, custom Lambda functions were required to trigger the AWS Glue crawler and retrieve the results from the first data-split task so that the appropriate size batch job could be provisioned.

As the fields used for splitting the data were the same as those used for blocking on each node, the comparison space was not different from running a linkage of the entire data set on a single machine. With the same comparison space and probabilistic parameters, the accuracy of the linkage is also identical. Having a mechanism for distributing linkage processing on multiple nodes with no reduction in accuracy is certainly a massive advantage for data linkage units looking to extend their linkage capacity.

The AWS Batch job definition’s retry strategy was configured with five attempts, applying to each job in the batch. This provides some resilience to instance failures, outages, and failures triggered within the container. However, in our evaluation, this feature was never triggered. The timeout setting was set to a value well beyond what was expected as jobs that time out are not retried, and our prototype did not handle this particular scenario. Although our implementation of the step function provided no failure strategies for any task in the workflow, handling error conditions is supported and retry mechanisms within the state machine can be created as desired. An operational linkage system would require these failure scenarios to be handled.

Improvements to the prototype will address some of the other limitations found in the existing implementation. For example, S3 data transfer times could be reduced by using a series of smaller result files for pairs and uploading all of these in parallel. The over-matching and duplication of pairs could be addressed by improving the indexing algorithm used to split data. Although there is inevitably going to be some overlap of blocks, our naïve implementation could be improved. Our algorithm for distributing blocks attempts to distribute workload as evenly as possible based on the estimated comparison space. Discarding overly large blocks helps prevent excessive load on single matching nodes. However, it relies on secondary blocks to match the records within and only partly prevents imbalanced load distribution. The block-based load balancing techniques developed for the MapReduce linkage algorithms can be applied here to mitigate data skew further, where record pairs are distributed for matching instead of blocks.

As improvements to PPRL techniques are developed over time, these changes can be factored in with little change to the model. Future work on the prototype will look to extend the capability of PPRL to use additional security advances such as homomorphic encryption [42] and function-hiding encryption [43].

### Conclusions

The model developed and evaluated here successfully extends linkage capability into the cloud. By using PPRL techniques and moving computation into cloud infrastructure, privacy is maintained while taking advantage of the considerable scalability offered by cloud solutions. The adoption of such a model will provide linkage units with the ability to process increasingly larger data sets without impacting data release protocols and individual patient privacy. In addition, the ability to store detailed linkage information provides exciting opportunities for increasing the quality of linkage and advancing the analysis of linkage outputs. Rich analytics, machine learning, automation, and visualization of these additional data will enable the next generation of quality assurance tooling for linkage.

## Abbreviations

API: application programming interface
AWS: Amazon Web Services
CPU: central processing unit
GPU: graphics processing unit
LSH: locality sensitive hashing
PPRL: privacy-preserving record linkage
TTP: trusted third party
